# Enhancing Gel-Based Drilling FIuids for Oil Sands Recovery Using Nitrogen-Doped Carbon Quantum Dots as AsphaItene Dispersants

**DOI:** 10.3390/gels11120942

**Published:** 2025-11-24

**Authors:** Weichao Du, Xueqi Feng, Yi Zhang, Wei Wang, Wenjun Shan, Le Xue, Gang Chen

**Affiliations:** 1 Engineering Research Center of Oil and Gas Field Chemistry, Xi’an Shiyou University, Xi’an 710065, China; duweichao@xsyu.edu.cn (W.D.); 24212071161@stumail.xsyu.edu.cn (X.F.); gangchen@xsyu.edu.cn (G.C.); 2 State Key Laboratory of Deep Earth and Mineral Exploration, Chinese Academy of Geologcial Sciences, Beijing 100037, China; wangwei1992@cags.ac.cn; 3 Oil & Gas Survey Center, China Geological Survey, Beijing 100083, China; swenjun@mail.cgs.gov.cn; 4 School of Petroleum Engineering, China University of Petroleum (Huadong), Qingdao 266580, China; bz25020011@s.upc.edu.cn; 5 Shaanxi Province Key Laboratory of Environmental Pollution Control and Reservoir Protection Technology of Oilfields, Xi’an Shiyou University, Xi’an 710065, China

**Keywords:** carbon quantum dots, asphaltene dispersion, gel-based drilling fluid, oil sands recovery

## Abstract

Oil sands drilling frequently contaminates water-based xanthan gels with highly viscous asphaltenes, collapsing their three-dimensional network and causing barite sag, high fluid loss and poor cuttings transport. Nitrogen-functionalized carbon quantum dots (N-CQDs) were hydrothermally synthesised from citric acid and 1-hexadecylamine and characterised by means of FT-IR, TEM and TGA. The concentration-dependent influence of N-CQDs (0–1.2 wt%) on gel viscoelasticity, microstructure and filtration properties was evaluated through rheometry, API and fluid-loss tests. At 0.01 wt% N-CQDs, the viscosity of the adsorbed oil phase dropped by 50% and the mean droplet diameter decreased from 247.7 µm to <100 µm. Consequently, the xanthan gel exhibited a significant enhancement in its mechanical strength and fluid loss performance. Wax-crystal growth was simultaneously inhibited, lowering the pour point by 6 °C. N-CQDs act as nanospacers that disrupt π-stacking of asphaltenes and hydrogen-bond to the polymer backbone, thereby restoring gel strength and sealing capacity. The work provides a sustainable, low-toxicity route to rejuvenate gel-based drilling fluids contaminated by heavy oil and facilitates their reuse in oil sands reservoirs.

## 1. Introduction

As an important unconventional oil resource, oil sands play a crucial role in modern energy production. Globally, Alberta, a province in Canada, is currently the largest region containing oil sands [1]. The distribution of remaining recoverable reserves of heavy oil and tar sands is uneven [2]. In light of the escalating energy crisis worldwide, the expansion of crude oil sand resources is poised to become an essential supplement to conventional petroleum reserves [3]. However, the development of abundant bitumen from oil sand reservoirs faces significant challenges due to the unique physical properties of this high-viscosity, multi-phase system [4]. Bitumen exhibits extremely low fluidity; its high viscosity greatly impedes normal drilling activities within oil sand reservoirs [5]. During the drilling process, bitumen frequently invades the wellbore along with the drilling fluid [6,7,8,9]. The interaction between bitumen and drilling fluid can alter the latter’s characteristics, and when bitumen invades beyond a critical threshold, paste-like flocs form, indicating severe contamination. Various complex issues arise at drilling sites, including contamination, loss of circulation, well instability, and stuck pipes [10]. Oil sands exhibit plastic and creep properties, with the total content of resin and asphaltene reaching up to 80%. The π-π stacking effect between condensed rings leads to the formation of stable asphaltene clusters and high apparent viscosity, which is advantageous for oil sand development. However, it can also cause complex problems such as annular plugging or pipe sticking, making it difficult to filter out material with solid control equipment and thereby hindering drilling fluid circulation.

In this context, the role of gel-based drilling fluids becomes critical. These fluids, often structured by biopolymers like xanthan gum into a three-dimensional hydrogel network, are essential for suspending cuttings, controlling filtration, and maintaining wellbore stability. The invasion of bitumen can disrupt this delicate gel structure, compromising its rheological and filtration properties. Asphaltenes can adsorb onto various carbon nanomaterials, including reduced graphene oxide (rGO), carbon nanotubes (CNTs), and activated carbon [11]. Recently developed green nanocomposites, synthesized using safe materials such as nanoparticles (NPs), also have the ability to bind with asphaltene molecules, stabilizing them and preventing self-assembly and deposition as nanoaggregates [12]. These nanoparticles possess unique structural properties, including a large surface area [13], which enhances their asphaltene adsorption capabilities. Carbon Quantum Dots (CQDs), as a novel class of carbon nanomaterial, have garnered significant attention due to their excellent physicochemical properties and wide-ranging application potential [14]. Although research is still limited, CQDs demonstrate notable advantages in inhibiting asphaltene aggregation due to their small size [15,16], strong chemical stability, low toxicity, environmental friendliness, and ease of surface modification [17,18]. While solidifiers and emulsifiers may offer positive effects under certain conditions, numerous practical challenges limit their broader application [19,20]. Therefore, techniques focused on chemical dispersion and viscosity reduction, particularly those involving the development of novel bitumen dispersants that are compatible with and enhance gel-based systems, may hold significant promise.

**Figure 1:**
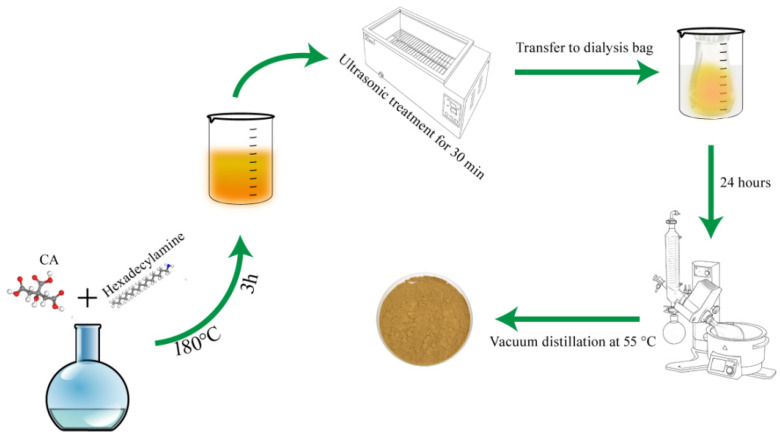
Synthesis process of CQDs.

This pioneering study investigates the use of CQDs as asphaltene dispersants within a gel-based drilling fluid system, utilizing functionalized CQDs to reduce heavy oil viscosity. Nitrogen was introduced using 1-hexadecylamine as the carrier, while citric acid served as the carbon donor (Figure 1). The long carbon chain in 1-hexadecylamine enhances hydrophobic properties and improves binding capacity with asphaltene [21]. Currently, no established evaluation methodologies exist for asphaltene dispersants in gel environments. This study primarily assesses efficacy through high-temperature rolling experiments and viscosity measurements, followed by formulation optimizations. Furthermore, the research aims to identify a suitable formulation that meets practical engineering requirements, potentially enhancing heavy oil extraction rates and broadening drilling fluid mobility by preserving the integrity of the gel structure under challenging conditions.

## 2. Results and Discussion

### 2.1. Characterization Results

#### 2.1.1. FT-IR Analysis

**Figure 2:**
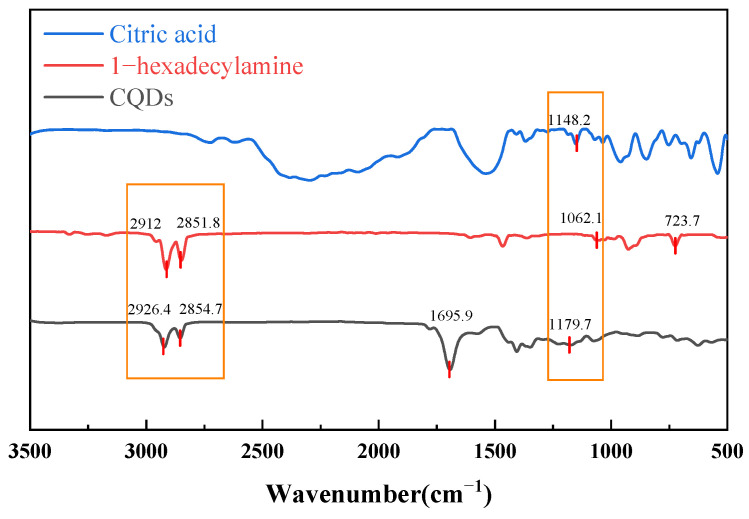
Infrared spectra of CQDs, citric acid and 1-hexadecylamine.

The infrared spectral analysis of the sample, depicted in Figure 2, reveals critical insights into its molecular structure. Key absorption bands indicate the presence of specific functional groups, confirming the compound’s characteristics.

The analysis highlights two distinct absorption doublets at 2851.8 cm^−1^ and 2926.4 cm^−1^, attributed to the C-H antisymmetric and symmetric stretching vibrations of the methylene (-CH_2_-) groups in the aliphatic chain. A notable peak at 1695.9 cm^−1^ corresponds to the stretching vibration of the carbonyl group (C=O), slightly below the typical value of 1700 cm^−1^, likely due to conjugation or hydrogen bonding effects. Additionally, multiple peaks at 1148.2 cm^−1^ and 1179.7 cm^−1^ indicate C-O stretching vibrations, suggesting the presence of alcohol, ether, or ester functional groups. The peak at 723.7 cm^−1^ relates to the planar rocking vibration of (CH_2_)_n_ groups, indicating a long linear aliphatic chain. Collectively, these spectral characteristics confirm that the compound features an extended aliphatic structure alongside a conjugated framework, aligning with the structural properties of CQDs.

#### 2.1.2. Absolute Fluorescence Quantum Yield

**Figure 3:**
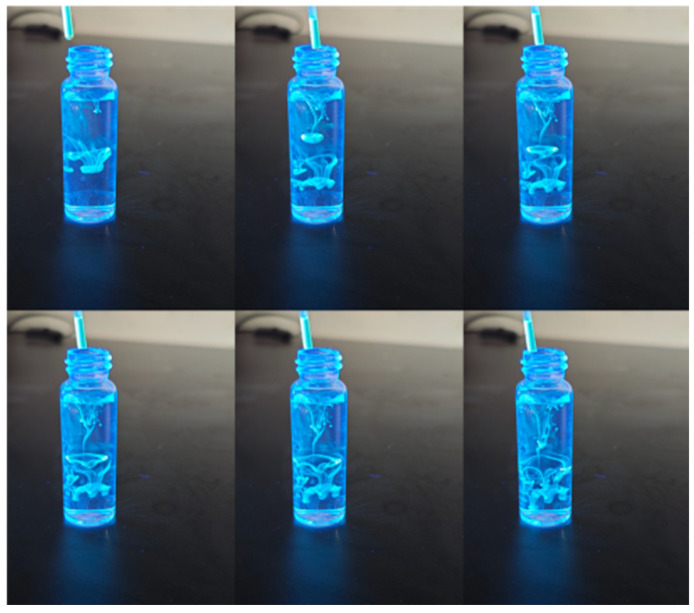
Optical behavior of CQDs under UV light.

As illustrated in Figure 3, carbon quantum dots (CQDs) exhibit remarkable optical properties, characterized by their ability to emit vibrant fluorescence when exposed to ultraviolet light.

**Figure 4:**
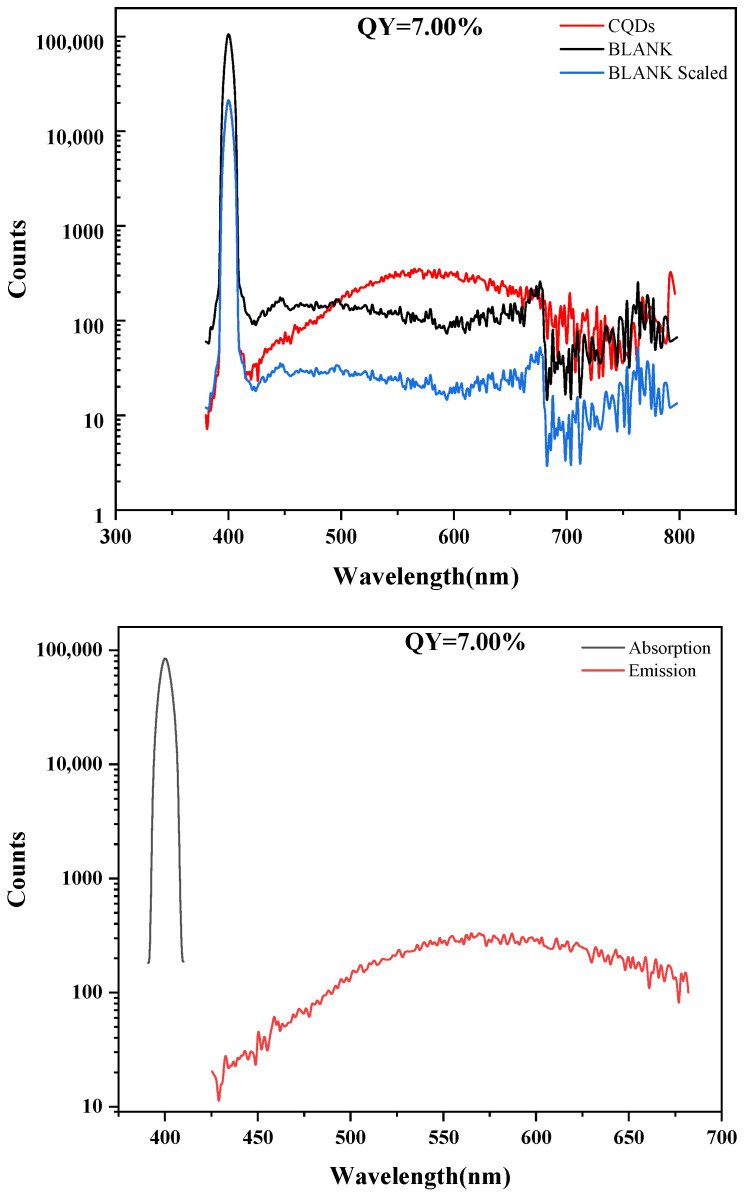
Absolute fluorescence quantum yield of CQDs.

As shown in Figure 4, the fluorescence quantum yield (QY) of the carbon quantum dots (CQDs) was determined to be 7.00%. This result was obtained based on precise parameter settings and background correction: to mitigate the contribution from the intense Rayleigh scattering peak around 400 nm, its spectral region (391.0–410.0 nm) was defined as the scattering interval and subtracted; the emission integration range was set from 425.0 to 682.0 nm to cover the effective emission of the CQDs between 425 and 700 nm, while prudently excluding the noisy region beyond 700 nm where the signal-to-noise ratio deteriorates significantly. The overall emission signal of the sample was relatively weak. A QY of 7.00% falls within the moderate-to-low range typical for CQDs. This outcome clearly indicates that the synthesized CQDs possess definite fluorescent properties, but there remains substantial room for improving their luminescence efficiency. The relatively low QY is commonly attributed to factors such as defect-state recombination within the carbon core, competition from non-radiative transitions associated with surface functional groups, and the specific energy level structure.

#### 2.1.3. TEM

**Figure 5:**
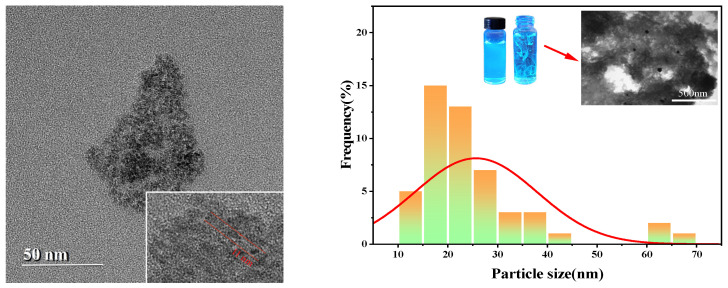
Particle size distribution histogram of CQDs.

The transmission electron microscope (TEM) analysis shows that the carbon quantum dots are uniformly distributed with a minimum size of about 11.0 nm and an average size of 25.66 nm. As shown in Figure 5, this size distribution is larger than the commonly observed size distribution of carbon dots.

TEM images show clear carbon quantum dot morphology, confirming their uniform dispersion in the sample. The minimum size of about 11.0 nm indicates a consistent formation process, while an average size of 25.66 nm points to a significant increase compared to conventional carbon dots. This large size may affect the optical and electronic properties of quantum dots, potentially enhancing their applicability in various fields. These findings highlight the unique properties of these carbon quantum dots and their potential for further research and applications.

#### 2.1.4. TGA

**Figure 6:**
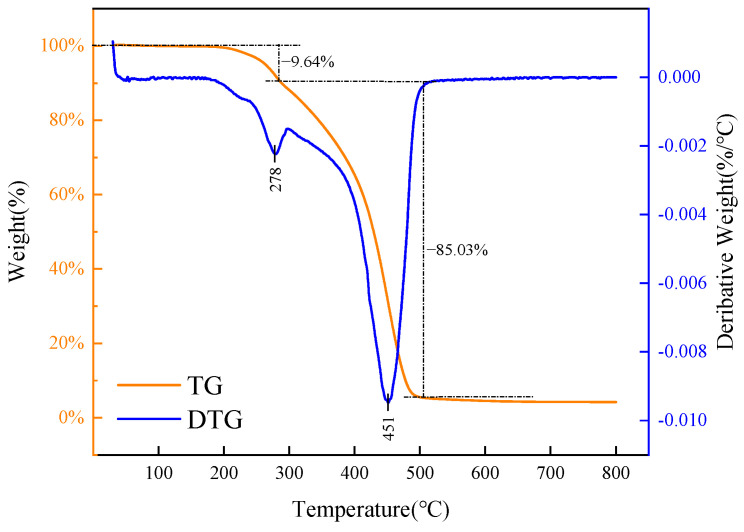
Thermogravimetric analysis of CQDs.

Figure 6 presents the thermogravimetric curve of carbon quantum dots (CQDs), illustrating their thermal stability and decomposition behavior. Understanding these characteristics is crucial, as high temperatures can compromise the structure and functionality of CQDs, particularly affecting their role as asphalt dispersants.

The analysis of the thermogravimetric curve reveals three distinct stages of weight loss for the CQD sample. Initially, from 100 °C to 200 °C, there is minimal mass loss, indicating the carbon dots’ resilience to high temperatures. In the second stage, between 200 °C and 285.8 °C, the sample experiences a relatively rapid weight loss of 9.64%, likely due to the gasification of unreacted raw materials or the decomposition of unstable impurities, as this range nears the boiling point of the reactants. The most significant weight loss occurs in the third stage, from 285.8 °C to 506.5 °C, where an 85.03% reduction is observed. This drastic decrease can be attributed to the instability of molecular chains within the CQDs at elevated temperatures, leading to the breakdown of the main chain and the formation of low molecular weight hydrocarbons. Beyond 506.5 °C, the thermogravimetric curve stabilizes, indicating the conclusion of the thermal decomposition process, with a total weight loss of 95.78% for the sample.

### 2.2. Performance Analysis

#### 2.2.1. Evaluation of Viscosity Reduction Performance

**Figure 7:**
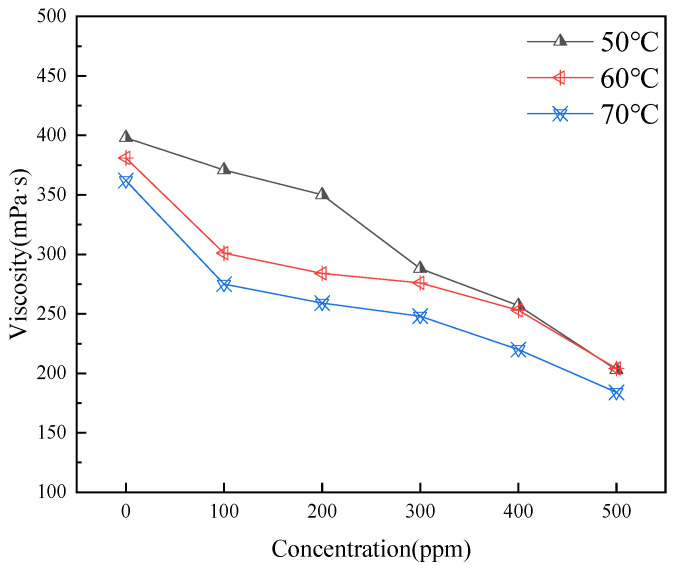
Effect of CQD addition on viscosity at different temperatures.

Figure 7 illustrates that the addition of carbon quantum dots (CQDs) generally leads to a reduction in the viscosity of the oil sample. This effect is particularly pronounced at higher initial viscosities, although the rate of viscosity reduction begins to diminish as the dosage increases. Each test was conducted in triplicate (*n* = 3) and averaged.

The mechanism behind this viscosity reduction can be attributed to the ability of smaller CQDs to intercalate within asphaltene aggregates, disrupting their layered structure. The surfaces of CQDs possess hydrogen-bonding sites, such as -OH groups, which interact with asphaltene molecules, effectively regulating the intermolecular forces within the dispersion. Notably, when the CQD concentration reaches 0.05 wt%, a significant further reduction in viscosity is observed. This enhanced effect is likely due to the higher concentration of CQDs improving the system’s homogeneity, resulting in better separation of the asphaltenes present in the crude oil. This interaction not only facilitates a decrease in viscosity but also enhances the overall stability of the dispersion.

#### 2.2.2. Adhesion Rate Following High-Temperature Aging

**Figure 8:**
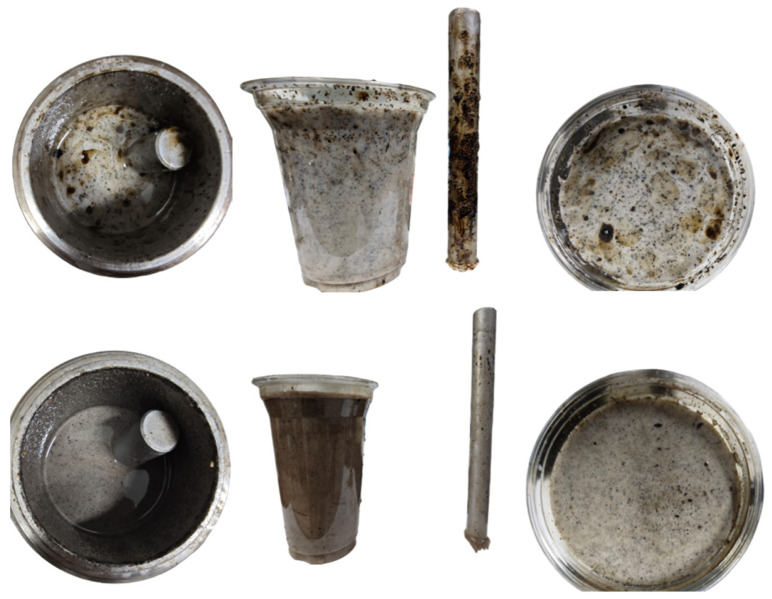
Comparison of the blank sample and 0.01% dosage group.

In Figure 8, from left to right, the images illustrate the opening of the tank after aging, the adhesion of the steel bar, and the dispersion of the oil droplets. The first row displays the blank sample, while the second row displays the results obtained with a 0.01% additive.

As shown in Figure 8, after hot rolling aging, the initial base slurry shows obvious problems of uneven dispersion and serious adhesion. After opening the tank, the liquid level is turbid and the oil droplets are coarse. When dumped into a transparent plastic cup, it can be seen that the oil droplets are mostly concentrated in the upper part, and the color of the lower slurry is shallow, indicating that the oil sand has floated and separated. In addition, a large amount of oil sand is attached to the surface of the right steel bar, which further indicates that the adhesion of the slurry is enhanced and the dispersion stability is poor after aging.

In contrast, after adding dispersants, the slurry state is significantly improved. The oil droplets are uniformly dispersed in the slurry, the particles are fine, the overall base slurry color is deep and uniform, and there is no obvious stratification. The oil sand adhering to the surface of the steel bar is also significantly reduced, indicating that the dispersant effectively improves the dispersion and stability of the system and reduces the adhesion tendency of oil sand. In summary, by using fine sand and dispersant, the dispersion state of oil sand can be significantly improved, the adhesion to the metal surface can be reduced, and the uniformity and stability of the overall system can be improved.

In order to understand the effect of CQD concentration on the adhesion rate more accurately, further experiments were carried out at different temperatures and concentrations. Each test was conducted in triplicate (*n* = 3) and averaged.

**Figure 9:**
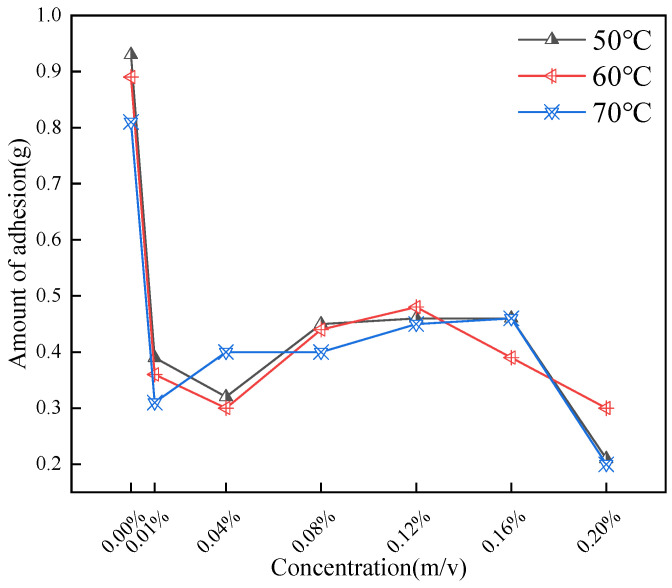
Impact of varying concentrations and temperatures on adhesion.

As shown in Figure 9, the addition of CQDs effectively reduces the adhesion of oil sand to the steel bar. At 0.01% CQD dosage, the decrease is the most obvious, which is due to the adsorption of CQDs on oil sand particles and the decrease of aggregation between small particles. As the concentration of CQDs continued to increase, the amount of adhesion was further reduced. When the adhesion reached 0.08%, it began to increase slightly and tended to be stable. It was speculated that the dispersion of CQDs in oil sand reached the threshold at this time. At excessive CQD loadings, competitive adsorption on asphaltene surfaces leads to crowding of alkyl chains, which sterically hinders further intercalation. As the CQD concentration increases beyond this point, the measured adhesion drops again: abundant carbon nanoparticles (CQDs) encapsulate the asphaltenes, forming a robust hydrophobic barrier that effectively isolates the aggregates.

#### 2.2.3. Drilling Fluid Rheology

**Figure 10:**
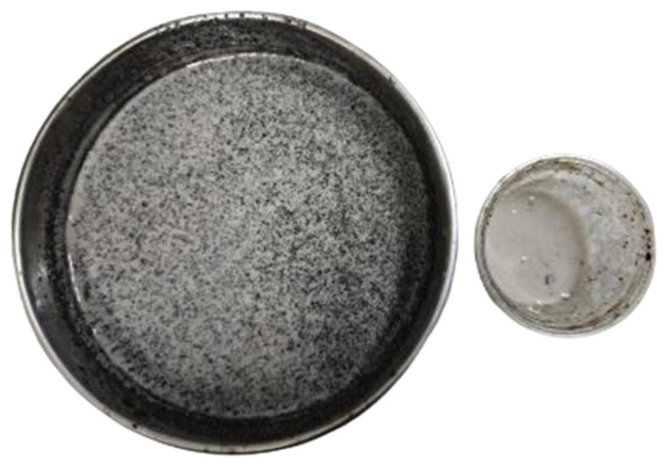
Screening of base slurry through mesh.

**Figure 11:**
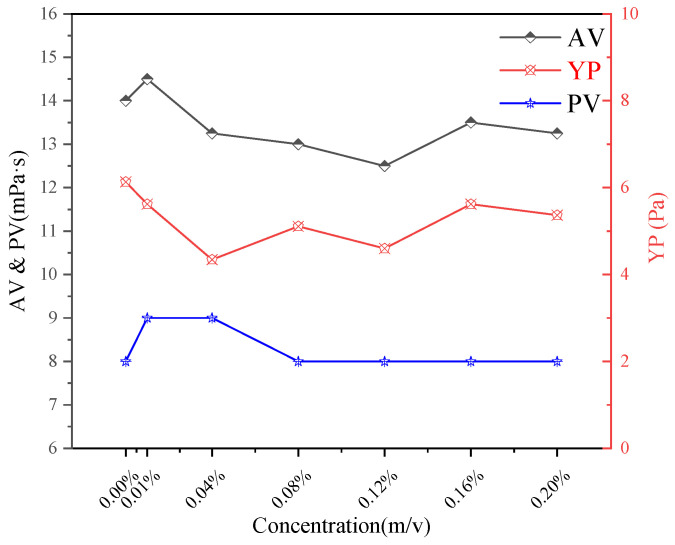
Impact of CQDs on the fluidity of drilling fluids at 50 °C.

The base slurry after hot rolling aging is filtered for subsequent experiments. Figure 10 shows the drilling fluid base slurry passing through the screen. Figure 11 shows the changes of apparent viscosity (AV), plastic viscosity (PV) and yield point (YP) of drilling fluid with CQD addition. Each test was conducted in triplicate (*n* = 3) and averaged.

With the increase of CQD concentration, the AV value of drilling fluid decreased slightly, and reached the lowest at the dose of 0.16%. The PV increased slightly at low concentration, and then returned to the initial value. The change of YP showed a trend of decreasing first and then increasing, and reached the lowest value at 0.04%. The yield point (YP) is a key indicator of the gel strength and the three-dimensional network structure within the drilling fluid gel. The initial decrease in YP with low CQD addition suggests a mild disruption of the native gel structure by the nanoparticles, potentially breaking some weak cross-links. However, as concentration increases, the CQDs may begin to participate in or reinforce the gel network, leading to a recovery and then an increase in YP. In general, the addition of CQDs has a significant effect on the fluid properties of drilling fluid, especially in the low concentration range, where the changes of AV and YP are more obvious, while PV is relatively stable. These changes indicate that CQDs can optimize the rheological properties of drilling fluids and modulate the gel strength by adjusting its concentration.

#### 2.2.4. Filtration Loss

**Figure 12:**
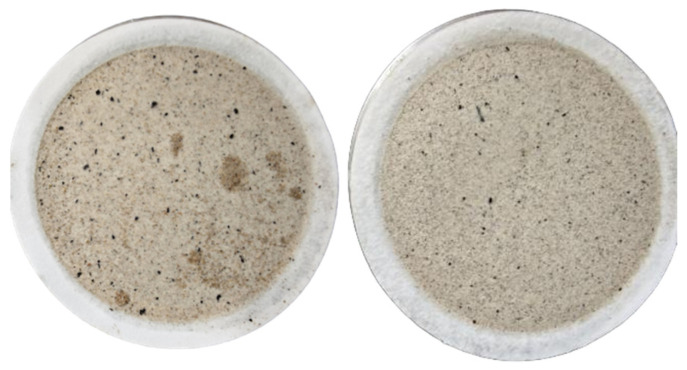
Blank filter cake (**left**); filter cake with dispersant addition (**right**).

**Figure 13:**
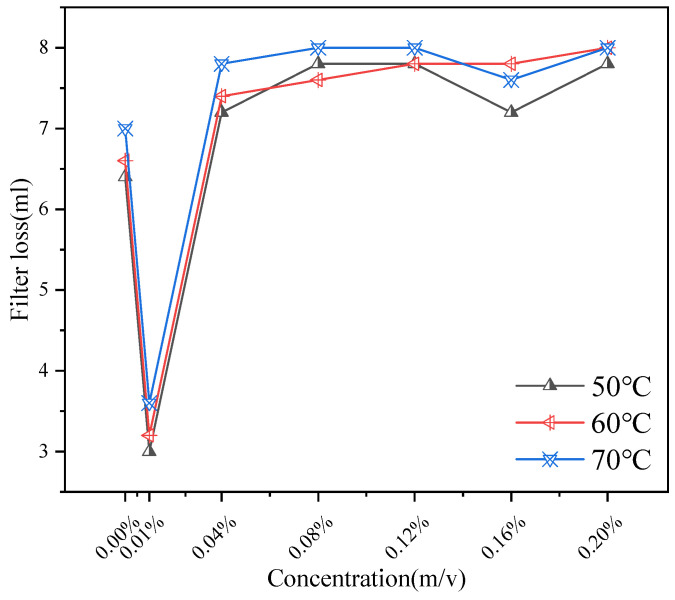
The Impact of CQDs on API Filtration.

The filter cake corresponding to the blank sample on the left has larger oil droplets and an uneven color. In contrast, after adding the dispersant, the filter cake particles are more evenly dispersed and smaller (Figure 12). This improved microstructure of the filter cake, which is a compressed gel layer, indicates that CQDs help form a more compact and less permeable gel barrier, enhancing filtration control. Figure 13 shows the effect of CQDs on filtration at different dosages and temperatures.

As shown in Figure 13, the effect of addition on API filtration shows a trend of inhibition first, then promotion and inhibition again. Measurements were performed according to API RP 13B-1. Each test was conducted in triplicate (*n* = 3) and averaged. When the concentration is 0.01%, the filtration rate drops sharply to about 3 mL, indicating that the additive has a significant inhibitory effect on the filtration rate in this concentration range. In the concentration range of 0.01~0.12%, the filtration rate increased gradually and reached the maximum value at 0.12%. The change of filtration rate from 0.12% to 0.20% indicates that the filtration is inhibited when the concentration increases to a certain extent. This non-monotonic behavior can be attributed to the dual role of CQDs: at optimal concentrations, they disperse asphaltenes and fine solids, promoting a dense gel cake; at intermediate concentrations, they might slightly interfere with the gel network’s ability to form a seamless barrier; and at higher concentrations, they themselves contribute to building a robust, nanoparticle-reinforced gel structure that effectively reduces fluid loss.

#### 2.2.5. Dispersion Properties

**Figure 14:**
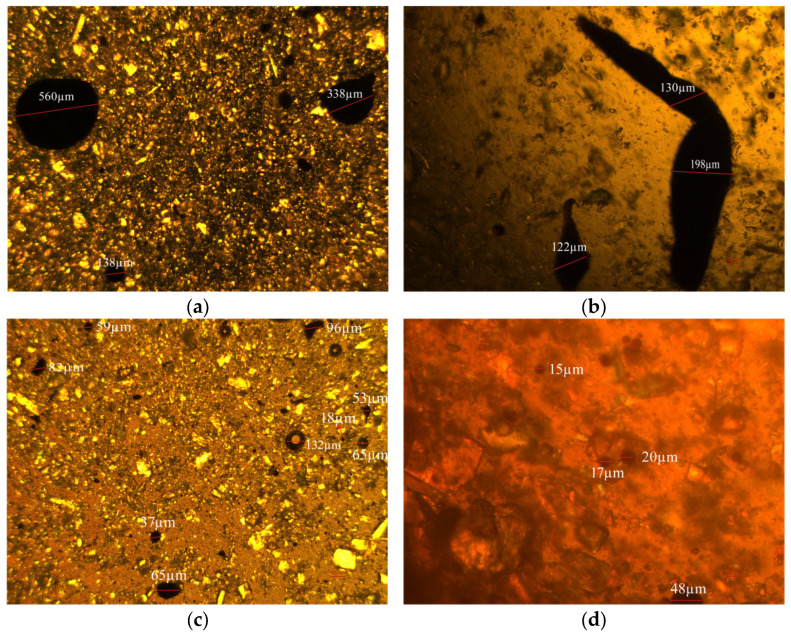
Dispersion analysis: blank samples ((**a**,**b**), scale bars: 100 µm) vs. 0.1% CQDs samples ((**c**,**d**), scale bars: (**c**) 100 µm; (**d**) 25 µm).

Figure 14 illustrates the dispersion of oil droplets containing 0.01% carbon quantum dots (CQDs). Notably, the average size of the oil droplets significantly decreases from 247.7 µm to under 100 µm with the addition of CQDs, highlighting a marked improvement in dispersion.

In detail, the introduction of CQDs alters the interaction dynamics within the oil matrix. The hydroxyl and amine groups present on the CQDs establish new hydrogen bonds with the oxygen- and nitrogen-containing groups found in asphaltenes, effectively disrupting the existing hydrogen-bond network. Additionally, the alkyl chains on the CQDs create a nonpolar interfacial layer that mitigates polar interactions and reduces π-π stacking. This dual action facilitates the dispersion of asphaltenes into smaller, less tightly associated aggregates, enhancing the overall stability of the oil droplets.

Traditional strategies rely on synthesizing polymers with increasingly complex architectures. The terpolymers, incorporating two or more types of polar functional groups, can achieve higher rates, often requiring synergistic combinations of different viscosity reducers for optimal effect [22]. This pathway necessitates intricate molecular design and complex formulation processes.

In stark contrast, the CQDs developed in this work demonstrate a fundamentally different and simplified approach. The most significant advantage lies in the substantially lower dosage required for our CQDs compared to conventional polymers. This high efficiency at low concentrations can be attributed to the unique nano-scale properties of CQDs: their ultra-small size and large specific surface area facilitate better dispersion and penetration into the heavy oil network, while their abundant surface functional groups effectively disrupt the associative structures of asphaltenes and resins. Furthermore, the carbonaceous nature of CQDs suggests a superior environmental profile compared to synthetic polymers.

### 2.3. Mechanism Analysis

#### 2.3.1. Morphological Characteristics of Wax Crystals

**Figure 15:**
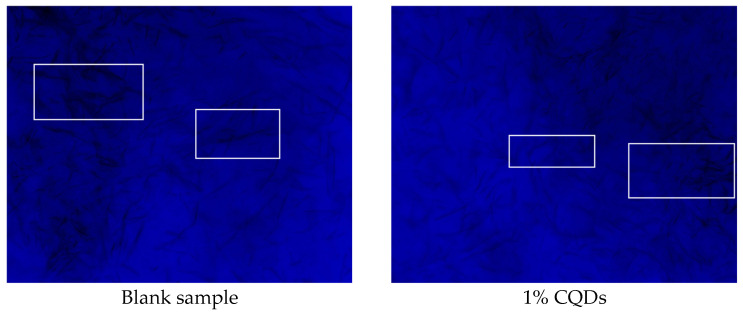
Morphological characteristics of wax crystals.

Figure 15 displays the original morphology of wax crystals observed under polarized light microscopy with a 5× objective, revealing a typical crystal structure. Upon the addition of 1% carbon quantum dots (CQDs), the wax crystals become notably finer and more uniformly dispersed, as shown in the white box of Figure 15.

In detail, the presence of CQDs plays a significant role in altering the characteristics of wax crystals. The introduction of CQDs inhibits the growth and aggregation of the crystals, leading to a decrease in their size and promoting a more homogeneous distribution. This represents the most fundamental and prevalent viscosity-reduction mechanism. It aims to transform the originally network-prone, high-aspect-ratio acicular or platelet crystals into smaller crystals with more granular morphology, thereby promoting the dispersion of asphaltenes. For example, bio-based flow improvers derived from waste cashew nut shells offer a cost-effective and eco-friendly solution for waxy crude oil. They significantly enhance flow assurance by modifying wax crystal morphology, which effectively reduces the pour point and viscosity of the oil [23]. This improvement in microscopic morphology suggests that CQDs effectively disrupt the processes that contribute to wax crystal formation, enhancing the overall stability and performance of the wax-containing system. Changes in crystal size and melting onset indicate that CQDs inhibited wax crystal growth and nucleation.

#### 2.3.2. DSC

As shown in Table 1, the wax precipitation point increased by about 1.2 °C at very low concentrations (0.1%) and higher concentrations (≥1%), but the effect disappeared in the intermediate concentration range (0.4–0.8%).

**Figure 16:**
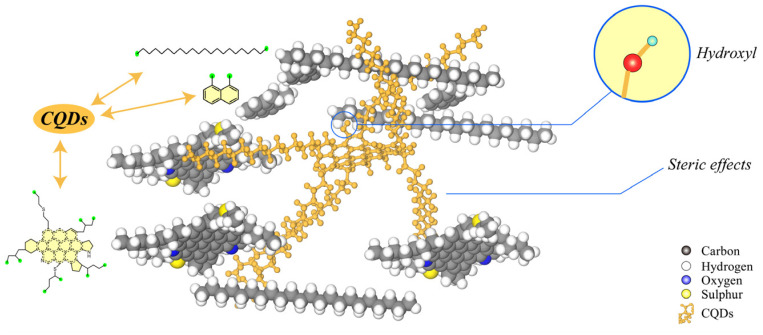
Dispersion Mechanism of CQDs in Asphalt.

Overall, the addition of CQDS does not significantly affect the wax precipitation point. As shown in Figure 16, CQDs disperse asphaltenes mainly through steric hindrance and the formation of new hydrogen-bonded structures.

## 3. Conclusions

In summary, nitrogen-doped carbon quantum dots (CQDs) were synthesized from citric acid and 1-hexadecylamine and evaluated as a potential asphaltene dispersant in gel-based oil sands drilling fluids. The CQDs were confirmed to possess the target chemical structure, optical properties, nanoscale size, and sufficient thermal stability. Performance tests demonstrated that the CQDs significantly reduce heavy oil viscosity, with a 50% decrease in the viscosity of the adsorbed oil phase achieved at a concentration of 0.05 wt%. The mean oil droplet diameter decreased from 247.7 µm to below 100 µm at a concentration of 0.01 wt%, considerably enhancing the dispersion stability within the gel system. The addition of CQDs also reduced oil sand adhesion to metal surfaces, improved the rheological properties and filtration control after high-temperature aging, and inhibited wax crystal growth, as evidenced by a 6 °C reduction in pour point. Mechanistic studies indicated that the CQDs function primarily as nanospacers that disrupt asphaltene π–π stacking and form hydrogen bonds with the polymer backbone, thereby restoring gel strength and sealing capacity. This study substantiates the potential of CQDs as a sustainable, low-toxicity additive for mitigating bitumen-related challenges in oil sands drilling, contributing to enhanced fluid performance and drilling efficiency. Further investigation into their long-term stability and field-scale application is recommended.

## 4. Materials and Methods

### 4.1. Materials

Citric acid (CA, solid, 99.5%) was purchased from Tianjin Zhonglian Chemical Reagent Co., Ltd. (Tianjin, China); 1-hexadecylamine (solid, 98%) was obtained from Yuanfeng Chemical Reagent Co., Ltd. (Shaoguan, China).

Sodium bisulfite (solid, 99.5%) was purchased from Nanjing Baimuda Biotechnology Co., Ltd. (Nanjing, China); Xanthan gum (Industrial grade) was obtained from China Oilfield Services Limited (Beijing, China); Modified starch (Industrial grade) was obtained from China Oilfield Services Limited; CaCO_3_ (Industrial grade) was purchased from Tianjin Damao Chemical Reagent Co., Ltd. (Tianjin, China).

### 4.2. Preparation of Drilling Fluid

The gel-based drilling fluid formulation is prepared via the sequential addition of components to fresh water. Initially, sodium bisulfite (0.7 wt%), xanthan gum (0.2 wt%, serving as the gelling agent to form a hydrogel network), and modified starch (0.75 wt%) are added under agitation at 12,000 rpm to promote pre-hydration and gelation. This is followed by the slow addition of calcium carbonate (8.6 wt%). Mixing is continued for 20 min to ensure homogeneous dispersion within the gel matrix and stable performance.

### 4.3. Preparation of CQDs

CQDs were prepared via solid-phase pyrolysis using citric acid as the carbon precursor and hexadecyl amine as the nitrogen source at a molar ratio of 1:3. The mixture was heated in an oven at 180 °C for 3 h. After the reaction, the resulting solid products were thoroughly ground to obtain a preliminary CQD powder. A measured amount of the powder was dissolved in anhydrous ethanol as the solvent. The mixture was ultrasonicated to achieve complete dissolution and form a homogeneous dispersion. The solution was filtered through a microporous membrane and then dialyzed in water using a 1 kDa molecular weight cut-off (MWCO) membrane for 24 h to remove impurities. The purified dispersion was transferred to a single-neck round-bottom flask and concentrated by rotary evaporation at 55 °C, yielding a bright yellow powder that was subsequently dried for further use.

### 4.4. Characterization of CQDs

#### 4.4.1. FT-IR

The synthesized CQDs were characterized by means of Fourier-transform infrared (FTIR) spectroscopy using a Bruker spectrometer (Bruker, Billerica, MA, USA). CQDs were purified with ethanol solvent, and the unreacted substances were removed by dialysis to obtain a powder sample. The CQD sample and potassium bromide (KBr) were homogenized through grinding at a 1:100 (*w*/*w*) ratio. The homogeneous mixture was then placed into the tableting mould to make a suitable sample for testing. Finally, the pressed sample film was put into the infrared spectrometer for the Infrared Spectrum Test. The scanning wavenumber range is 4000–500 cm^−1^. Reference spectra were similarly acquired for both citric acid and 1-hexadecylamine precursor materials.

#### 4.4.2. Absolute Fluorescence Quantum Yield

The absolute fluorescence quantum yield is determined using the integrating sphere method, which is a direct approach for measuring the absolute fluorescence quantum yield without the need for comparison with standard fluorescent materials. Its core principle is based on the conservation of photon energy. In the integrating sphere experiment, the photon count is equivalently calculated by measuring the luminous flux. Specifically, a standard white reflector is placed inside the integrating sphere as a reference surface, and the following three scenarios are measured separately. A blank reference spectrum: The sample cell containing pure solvent (the same as the sample solvent) is placed in the integrating sphere and irradiated with excitation light. This spectrum records all the unabsorbed excitation light and the scattered light from the solvent. The sample emission spectrum: The sample cell containing the test sample is placed in the integrating sphere and irradiated with the same excitation light. At this point, the spectrum includes the fluorescence emitted by the sample, the unabsorbed excitation light, and the scattered light from the solvent. The direct excitation spectrum: The sample cell is directly aligned with the detector, avoiding the inner wall of the integrating sphere. This spectrum is primarily used to determine the exact absorbance of the sample at the excitation wavelength, ensuring it falls within an appropriate low absorbance range to avoid inner filter effects.

By analyzing these spectra, the quantum yield can be calculated as the integrated area of the sample’s fluorescence emission spectrum divided by the equivalent area of the portion of excitation light absorbed by the sample. The latter can be obtained by comparing the difference between the integrated area of the excitation peak in the blank reference spectrum and the integrated area of the excitation peak in the sample emission spectrum.

#### 4.4.3. TEM

The CQD sample was dissolved in ethanol and sonicated for 30 min to form a homogeneous suspension. After drying at room temperature, the specimen was characterized using transmission electron microscopy (TEM), and high-resolution images of representative regions were obtained.

#### 4.4.4. TGA

The dried CQD powder (5–10 mg) was placed in an alumina crucible with nitrogen as the protective gas, and the temperature was raised from room temperature to 800 °C at 10 °C/min in a thermogravimetric analyzer. The thermal stability and component decomposition characteristics were analyzed.

### 4.5. Performance Evaluation

#### 4.5.1. Evaluation of Viscosity Reduction Performance

CQDs were introduced into the heavy oil sample in accordance with a concentration gradient, with concentrations set at 0.01 wt%, 0.02 wt%, 0.03 wt%, 0.04 wt%, and 0.05 wt%, respectively. Each concentration of the mixture is determined after full mixing. The viscosities of all heavy oil samples were measured by a rotational viscometer at 50 °C, 60 °C and 70 °C.

#### 4.5.2. Adhesion Rate Following High-Temperature Aging

In this part, the effect of aging temperature on rheological properties and filtration loss of the filtrate reducer was investigated. The base slurry consists of sodium bisulfite, calcium carbonate, modified starch and xanthan gum. Hot rolling at 50 °C, 60 °C and 70 °C for 1 h. The hot rolling sample comprises 30 g of simulated oil sand per 350 g of base slurry, with a gradual increase in the amount of CQDs.

#### 4.5.3. Drilling Fluid Rheology

A six-speed Rotational viscometer was employed to measure the viscosity of the gel-based base slurry before and after hot rolling. The hot-rolled base slurry was filtered through an 80-mesh screen to remove large particle impurities.

#### 4.5.4. Filtration Loss

A filtration test was conducted on the gel-based drilling fluid base slurry sample following hot rolling treatment, and the measured data were compared with the filtration test results of an untreated blank control sample. The hot-rolled base slurry sample was placed in a standard API filter at a pressure of 0.69 MPa, and the filtrate loss was recorded over a period of 15 min to evaluate the stability of the gel filter cake.

#### 4.5.5. Dispersion Properties

Polarized light microscopy was employed to examine the morphological characteristics and microstructural changes of the oil droplets in the base slurry sample following thermal rolling aging treatment. The diameter and distribution of oil droplets prior to and following the addition of additives were compared by image analysis. In the experiment, the blank sample devoid of additives was systematically compared with the experimental group containing 0.01% additive to assess the impact of additive concentration on oil droplet behavior.

### 4.6. Mechanism Analysis

#### 4.6.1. Morphological Characteristics of Wax Crystals

The paraffin wax sample was heated to the molten state in a constant temperature water bath, with the temperature maintained for 20 min to ensure the uniformity and stability of the molten wax. Subsequently, CQDs were added to the experimental group and thoroughly stirred to ensure their uniform dispersion in the molten wax. The wax crystal morphology of a blank (without CQDs) was used as the control. The mixed wax liquid was slowly poured onto the clean glass slide and cooled at a low temperature for solidification. Upon complete solidification of the samples, the microscopic morphology characteristics of wax crystals were systematically observed and characterized using a microscope system equipped with a polarizing device.

#### 4.6.2. DSC

The wax precipitation temperature of oil samples with various additives was determined using Differential Scanning Calorimetry (DSC). DSC was run from 60 °C to −20 °C at a rate of 5 °C min^−1^ with a sample mass of 5 mg (Supplementary Material). Samples with varying concentration gradients, including blank oil samples without additives, were selected for testing.

## Figures and Tables

**Table 1:** Correlation between CQD concentration and wax precipitation temperature in heavy oil.

Concentration (%)	Temperature (°C)
0	53.5
0.1	54.7
0.4	53.5
0.8	53.5
1	54.7
1.2	54.7

## Data Availability

The original contributions presented in the study are included in the article; further inquiries can be directed to the corresponding author.

## Supplementary Materials

The following supporting information can be downloaded at: https://www.mdpi.com/article/10.3390/gels11120942/s1.
