# Supplementary material for: Enhancing Gel-Based Drilling FIuids for Oil Sands Recovery Using Nitrogen-Doped Carbon Quantum Dots as AsphaItene Dispersants
Source: Gels. 2025 Nov 24;11(12):942. doi: 10.3390/gels11120942 (PMC12732854; doi:10.3390/gels11120942)
Supplement: Supplementary file 1 [file gels-11-00942-s001.zip › gels-3963564-supplementary.pdf]

Section S1: Detailed Experimental Procedure for Differential Scanning Calorimetry (DSC)

1.1 Instrumentation
Instrument: Mettler-Toledo DSC 3
Software: STARe Software 14.00

S1.2 Experimental Parameters
Atmosphere: Inert gas
Method Name: "Wax Precipitation Point-2301"

S1.3 Detailed Procedure
Sample Preparation: A small, representative aliquot of the crude oil sample was accurately weighed into a standard aluminum DSC crucible. The crucible was hermetically sealed to prevent sample evaporation.
Temperature Program:
A dynamic cooling scan was initiated. The specific cooling rate is defined within the "Wax Precipitation Point-2301" method file used by the instrument.
Data Collection: The heat flow (Wg^{-1}) difference between the sample crucible and an empty reference crucible was continuously measured and recorded as a function of sample temperature (Ts) and reference temperature (Tr).

S1.4 Data Analysis and WAT Determination
The WAT is identified as the temperature at which the onset of a detectable exothermic deviation from the baseline occurs, corresponding to the crystallization of wax paraffins. As detailed in the primary data file, the WAT for this sample was determined by the instrument's software to be 53.52 °C.

S1.5 Data Accessibility
The complete raw data from this DSC experiment, including all recorded temperature and heat flow values for both the main and preliminary scans (curves !&]3[1 and !&]2[1), is provided in the accompanying data file. This allows for full transparency and independent verification of the result.

Section S2: Rheological Data Analysis and Calculation Method for Drilling Fluid

S2.1 Data Collection: Stress vs. Strain Rate

The fundamental rheological data is collected by measuring the shear stress (τ, in lb/100 ft^2) at various shear rates (γ, in s^{-1}). A standard protocol involves measuring the dial readings at different rotational speeds (RPM) of the viscometer, which are directly convertible to shear stress and shear rate, respectively.

S2.2 Summary

The following table summarizes the standard calculations:

Parameter	Formula
Apparent Viscosity (AV)	$AV=\theta_{600}/2$
Plastic Viscosity (PV)	$PV=\theta_{600}-\theta_{300}$
Yield Point (YP)	$YP=0.4788\times(\theta_{300}-PV)$

Where θ_{600} and θ_{300} are the dial readings from the rotational viscometer at 600 and 300 RPM, respectively.

This methodological approach ensures consistency with industry standards and allows for direct comparison of fluid performance.
